# Outbreak of Typhoid Fever in Children of Urban Vellore: A Report from the Surveillance for Enteric Fever in India Cohort

**DOI:** 10.4269/ajtmh.21-0593

**Published:** 2022-07-13

**Authors:** Manikandan Srinivasan, Kulandaipalayam Natarajan Sindhu, J. Senthil Kumar, Ranjith Kumar Ramasamy, Agila Kumari Pragasam, Pratheepa Aasaithampi, Venkata Raghava Mohan, Gagandeep Kang, Jacob John

**Affiliations:** ^1^ Wellcome Trust Research Laboratory, Division of Gastrointestinal Sciences, Christian Medical College, Vellore, Tamilnadu, India;; ^2^ Department of Clinical Microbiology, Christian Medical College, Vellore, Tamilnadu, India;; ^3^ Department of Community Health, Christian Medical College, Vellore, Tamilnadu, India

## Abstract

We report an outbreak of typhoid fever between April and June 2019 in the Surveillance for Enteric Fever in India cohort, a pediatric cohort from four contiguous semi-urban settlements of Vellore in South India. This cohort of children 6 months to 15 years of age was under surveillance from October 2017 to December 2019. A clustering of typhoid cases in the cohort was noted with reference to time, place, and person. The overall typhoid attack rate in the cohort was 0.9%, with the highest attack rate of 1.7% being documented in one of the four areas. The rate of hospitalization and complications in children who were typhoid positive during the outbreak was 28% and 2%, respectively. Given the background of suboptimal water, sanitation, and hygiene, and the risk of typhoid fever outbreaks in these settings, it is imperative that a typhoid vaccine be considered for introduction as a pragmatic preventive approach.

Typhoid fever caused by *Salmonella* Typhi, a bacillus exclusive to the human host, is known to occur in short, as well as long, transmission cycles.^1^ The short transmission cycle has been implicated in settings with *S*. Typhi endemicity through contamination of food or water in the immediate environment, where water, sanitation, and hygiene (WaSH) practices are suboptimal. Furthermore, outbreaks of typhoid fever can eventuate in these settings by long transmission cycles, where drinking water supplies of the community at large are contaminated with *S*. Typhi via the distribution system.^1^ The poorly engineered water supply and sewage systems in the urban areas of developing settings favor cross-contamination of drinking water with sewage, along with the contamination of food, putting the population—especially children—at high risk for typhoid.^2^^,^^3^ Based on the Surveillance for Enteric Fever in India (SEFI) cohort, we report an investigation conducted and the findings from an outbreak of typhoid fever in urban Vellore in 2019.

SEFI was established in October 2017 in four Indian settings—Delhi, Kolkata, Pune, and Vellore—to estimate the incidence of typhoid fever in children. The study details are described elsewhere.^4^ The Vellore cohort comprised ∼6,000 children between 6 months and 15 years of age from four contiguous semi-urban settlements of Vellore town—namely, Chinnallapuram, Ramnaickanpalayam, Kaspa, and Vasanthapuram. The cohort was under active surveillance for fever through weekly home visits by field research assistants and daily visits during fever episodes. Children with fever for 3 days or more met the study surveillance definition of suspected typhoid fever, and received a blood culture. Blood culture specimens were inoculated and processed using the BacT/ALERT automated system at the Department of Clinical Microbiology, Christian Medical College, Vellore, India. The antibiotic susceptibility testing of *S.* Typhi strains was done using the Kirby-Bauer disk diffusion method, with interpretation based on Clinical and Laboratory Standards Institute (CLSI) guidelines. Isolates were tested for ciprofloxacin sensitivity using minimum inhibitory concentration values determined by the standard broth microdilution method and interpreted using CLSI guidelines.

Between April and May 2019, a surge in typhoid fever cases was observed in the cohort, particularly in Kaspa. We confirmed this surge in cases as a result of an outbreak in the community by computing the number of typhoid cases reported between April and June 2019, which exceeded the mean + 2 SD cases reported during surveillance in the corresponding months of the previous years of 2017 and 2018.^5^^,^^6^ An outbreak investigation was thus conducted in the households of all four areas where the study children resided. A questionnaire was administered by the field research assistant, and information on demographic characteristics, source of drinking water, sanitation, history of consumption of unpackaged/unboiled milk and food consumed from street vendors (which included chutney, a local sauce made of grated coconut and water; raw salads; locally prepared flavored popsicles; flavored drinks; and fruit juices) was collected.

A case–control approach was used to study the risk factors associated with typhoid fever at the household level. A household with a case of culture-confirmed typhoid fever during the outbreak was designated as a case household; those households without cases were designated as a control household. Households from Kaspa that did not report a typhoid case in the past 2 months were chosen as control households. Unadjusted odds ratios and with 95% CIs were reported in the risk factor analysis. Stata 13 (StataCorp LLC, College Station, TX) was used for statistical analysis. A spot map of typhoid cases was plotted using geographic information system coordinates. Written informed consent was obtained from the head of the participating households. SEFI was approved by the Institutional Review Board of Christian Medical College (IRB min no. 10393).

During the outbreak between April and June 2019, 917 households were interviewed (within the SEFI cohort of 5,705 children from 3,075 households). On computation of the area-wise attack rates for typhoid fever, the rates in Chinnallapuram, Ramnaickanpalayam, Vasanthapuram, and Kaspa were 0.1% (2 of 1,527), 0.7% (10 of 1,516), 1% (8 of 838), and 1.7% (31 of 1,824), respectively, with Kaspa documenting the highest attack rate (Figure 1C). Cases documented outside the outbreak period were designated as sporadic cases. Fifty-one pediatric cases of blood culture–confirmed typhoid fever were reported from 44 households. The 44 households that had at least one case of culture-confirmed typhoid were designated as case households, with the remaining 873 as control households.

**Figure 1.**
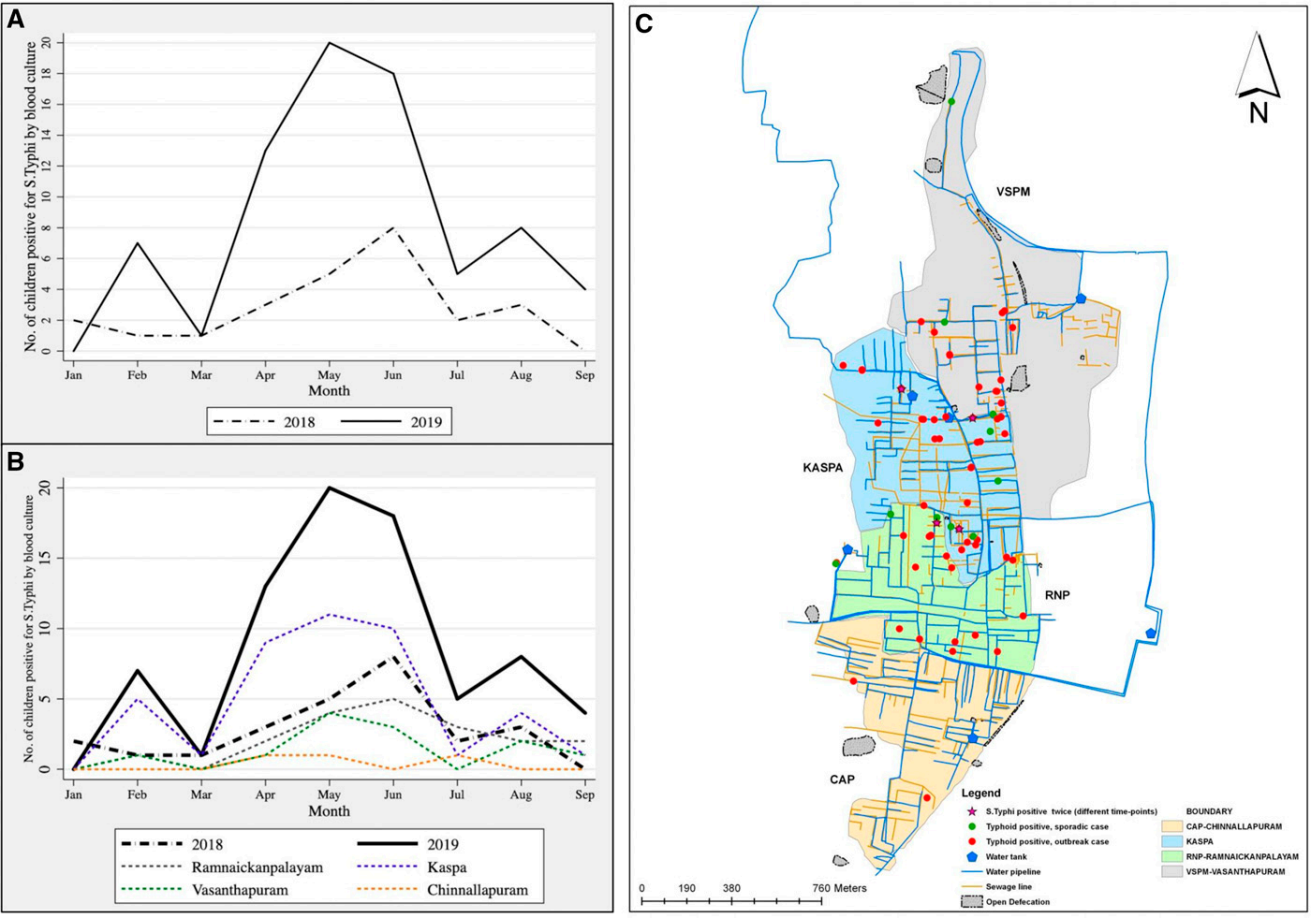
Epidemic curve showing the number of children with typhoid fever in the Surveillance for Enteric Fever in India cohort between January and September 2018 and 2019 overall (**A**) and area-wise in 2019 (**B**). (**C**) Spot map showing cases of typhoid fever in the four urban areas of Vellore during 2019 indicating the sporadic and outbreak cases using a geographic information system. This figure appears in color at www.ajtmh.org.

Of the 51 typhoid cases, 25 cases (49%) were reported in children 5 to 10 years old. Hospitalization was observed at a rate of 27.5% (14 of 51) among the typhoid cases. Subsultus tendinum, a rare neurological complication of typhoid fever, was noted in a 5-year-old child (rate of overall complications, 2%). The child recovered with treatment of typhoid fever and anti-seizure medications, with no residual sequelae (Table 1).^7^

**Table 1:** Baseline characteristics of the children with typhoid fever during the outbreak in the Surveillance for Enteric Fever in India cohort (*N* = 51)

Characteristic	*n* (%)
Age group, years	
< 5	9 (17.7)
5–10	25 (49)
10–15	17 (33.3)
Gender	
Male	28 (54.9)
Female	23 (45.1)
Area-wise attack rates (per 100 children)	
Chinnallapuram	2 of 1,527 (0.1)
Ramnaickanpalayam	10 of 1,516 (0.7)
Vasanthapuram	8 of 838 (1.0)
Kaspa	31 of 1,824 (1.7)
Clinical features	
Fever	51 (100)
Headache	28 (54.9)
Cough	22 (43.1)
Vomiting	21 (41.2)
Abdominal pain	20 (39.2)
Diarrhea	13 (25.5)
Constipation	1 (2)
Blood in stool	1 (2)
Jaundice	1 (2)
Duration of fever, days; median (IQR)	8 (7–10)
Highest temperature, °F; median (IQR)	103 (102–103.6)
Children with blood culture–confirmed typhoid fever who were hospitalized	14 (27.5)
Complication of subsultus tendinum	1 (2)
Antibiotic susceptibility testing	
Ampicillin sensitive	51 (100)
Chloramphenicol sensitive	51 (100)
Cotrimoxazole sensitive	51 (100)
Ceftriaxone sensitive	51 (100)
Azithromycin sensitive	51 (100)
Ciprofloxacin sensitive*	1 (2)

Of the 51 typhoid outbreak cases, 24 children received azithromycin for a median duration of 9 days; 11 received any two of the antibiotics- azithromycin, amoxycillin, chloramphenicol, ciprofloxacin, cefixime, ceftriaxone, and ofloxacin, for a median duration of 12 days; and 16 received any three of the antibiotics- azithromycin, cefixime, ceftriaxone, ciprofloxacin, amoxycillin, cefotaxime, gentamycin, ampicillin, doxycycline, linezolid, erythromycin, chloramphenicol, metronidazole, and cotrimoxazole, for a median duration of 15 days.

* Of the 51 isolates, only one isolate was susceptible to ciprofloxacin, with a minimum inhibitory concentration (MIC) of 0.06 μg/mL; 47 were of intermediate susceptibility, with an MIC ranging between 0.12 and 0.5 μg/mL; and three were resistant to ciprofloxacin, with an MIC ≥ 1 μg/mL.

Typical clustering of outbreak cases with reference to time, place, and person was observed. Clustering of 11 cases was noted within three contiguous streets of Kaspa. The majority of typhoid cases were in the month of May (Figure 1A). Furthermore, clustering of cases was noted within households, with five households reporting two or more cases. Three of these five households reported two cases of typhoid fever each, with secondary cases in these households being reported within 14 days from the onset of fever in the primary cases. The fourth household reported four cases serially in a month, following the onset of fever in the primary case. The fifth household had a child with relapse of typhoid fever, 21 days after the resolution of the previous episode, and was treated with intravenous ceftriaxone during both episodes. On bivariate analysis, the presence of a preschool child in the household and the consumption of raw salads such as onion/cucumber/tomato and locally prepared popsicles (water mixed with sugar and artificial color, sealed, and frozen in plastic covers) were associated significantly with the risk of contracting typhoid fever (Table 2).^8^ Whole genome sequencing of *S.* Typhi isolates during the outbreak showed that all 51 strains belonged to H58-lineage II (genotype-4.3.1.2).^9^ The phylogenetic analysis at the single nucleotide polymorphism (SNP) level revealed that 27 of 51 isolates (52.9%) belonged to a single strain with zero SNP differences, with the rest of the strains being diverse with SNP differences of 1 through 39. Of these 27 isolates belonging to a single strain, 21(77.7%) were isolated from children residing in Kaspa. Children in households that reported two or more typhoid cases during the outbreak were infected with the same *S.*Typhi strain with zero SNPs, except for the child in the household with four cases who was infected with a different strain (Supplementary Figure S1).

**Table 2:** Risk factors studied at the household level for typhoid fever (*N* = 917)

Risk factors	Households* (*N* = 917), n (%)	Households with typhoid fever (*n* = 44), n (%)	Unadjusted odds ratio (95% CI)
Source of drinking water†			
Unimproved	758 (82.7)	41 (93.2)	3.0 (1.0–15.2)
Improved	159 (17.3)	3 (6.8)	Ref.
Sanitation facility‡			
Unimproved	693 (75.6)	37 (84.1)	1.8 (0.8–4.1)
Improved	224 (24.4)	7 (15.9)	Ref.
Socio-economic status§			
Low	619 (67.5)	30 (68.2)	1.0 (0.5–2)
Middle	274 (29.9)	14 (31.8)	Ref.
High	24 (2.6)	0	–
Presence of preschool children in the household			
Yes	113 (12.3)	10 (22.7)	2.2 (0.9–4.7)
No	804 (87.7)	34 (77.3)	Ref.
Consumption of raw salads (onion/cucumber/tomato) (*n* = 909)			
Ever	493 (54.2)	30 (69.8)	2.0 (1.0–4.3)
Never	416 (45.8)	13 (30.2)	Ref.
Consumption of locally prepared popsicles			
Ever	328 (35.8)	24 (54.6)	2.2 (1.2–4.4)
Never	589 (64.2)	20 (45.5)	Ref.
Consumption of fruit juices (*n* = 898)			
Ever	335 (37.3)	13 (52)	1.9 (0.8–4.5)
Never	563 (62.7)	12 (48)	Ref.
Consumption of locally prepared flavored drinks (*n* = 898)			
Ever	523 (58.2)	16 (64)	1.3 (0.5–3.3)
Never	375 (41.8)	9 (36)	Ref.

Ref. = reference value.

* *N* = 917 households unless specified otherwise.

† Municipal water supply was classified as unimproved drinking water because of it being at risk of contamination before the point of collection at the household level.

‡ Households with toilets with its effluents being discharged into drains without treatment were classified as having unimproved sanitation. Those households with effluents from toilets contained in a septic tank were classified as having improved sanitation.

§ Socioeconomic status classification was done using modified Kuppuswamy scale that accounted for occupation, education, and selected assets.^8^

This outbreak of typhoid fever in our setting captured within the purview of SEFI, an active surveillance for fever in a pediatric cohort, saw the highest number of blood culture–confirmed typhoid cases in the 5- to 10-year age bracket. This finding from our setting is in agreement with the findings of the Surveillance for Enteric fever in Asia Project, in which typhoid cases were noted predominantly in children 5 to 10 years old.^10^ However, it is to be noted that there exists a considerable heterogeneity in typhoid disease burden within the Indian subcontinent with reference to age groups, and this needs to be factored in when planning typhoid vaccination strategies in high-risk settings.^10^

Hot climatic conditions prevail in Vellore from March to July. The average maximum temperatures recorded in May and June 2019 were 42°C and 39.2°C, respectively.^11^ This extreme weather condition perhaps plays a role in the accentuation of enteric fever cases, as a consequence of water scarcity, which prevails during the summer, especially in these densely populated settlements, hence limiting the availability of safe drinking water. An enhanced multiplication of *S.* Typhi in contaminated food, given high temperatures during the summer, probably adds further to the surge in enteric fever cases.^12^ Furthermore, high summer temperatures lead to an increased consumption of unsafe refreshments by children in these settlements, such as locally made flavored popsicles and flavored drinks, predisposing children to a high risk of enteric infections. A similar finding, in which typhoid fever was associated with the consumption of locally prepared drinks or popsicles, was reported from typhoid outbreak in Uganda as well as in an endemic setting in Chile.^13^^,^^14^

The rate of hospitalization and complications following typhoid fever in this outbreak was 28% and 2%, respectively. This is in contrast to the typhoid fever outbreak in Pakistan, where the rates documented for the same were 48% and 35%, respectively.^15^ A relatively lower rate of hospitalization and complications noted in our setting could be explained by the fact that the *S.* Typhi strains isolated from this setting are in general susceptible to first-line antibiotics, azithromycin as well as ceftriaxone with the exception of ciprofloxacin, which was not the case in the Extremely drug resistant (XDR) strain isolated in the outbreak at Sindh, Pakistan. Also, because the children in this outbreak were a part of the SEFI cohort and were under daily intensive follow-up during fever, initiation of the appropriate antibiotic was instituted at the earliest, as per the protocol.

The isolation of the same *S.* Typhi strain among the clustered cases at the household level indicates that these children could have acquired *S.* Typhi infection from the community or from the primary case in households. This highlights the importance of implementing a WaSH infrastructure toward achieving typhoid control in high-risk settings with suboptimal WaSH facilities. Furthermore, of the 51 outbreak cases, only two children (3.9%; overall SEFI cohort, 581 of 6,760 or 8.6%) had received a typhoid vaccine (the vaccine is not yet a part of the Indian national immunization schedule). This highlights the need to introduce the vaccine in these high-risk settings, and overall in the national immunization program.

To conclude, our investigation found that children from typhoid-endemic settings with suboptimal WaSH with poor vaccination coverage are at a high risk for typhoid fever. Improving WaSH in these resource-constrained settings is an enduring challenge. The risk of typhoid outbreaks in settings such as these in the Indian subcontinent necessitates the introduction of the typhoid conjugate vaccine as a priority.

## Supplemental Material


Supplemental materials
