# Supplementary figures and images for: Outbreak of Typhoid Fever in Children of Urban Vellore: A Report from the Surveillance for Enteric Fever in India Cohort
Source: Am J Trop Med Hyg. 2022 Jul 13;107(1):82–5. doi: 10.4269/ajtmh.21-0593 (PMC9294687; doi:10.4269/ajtmh.21-0593)

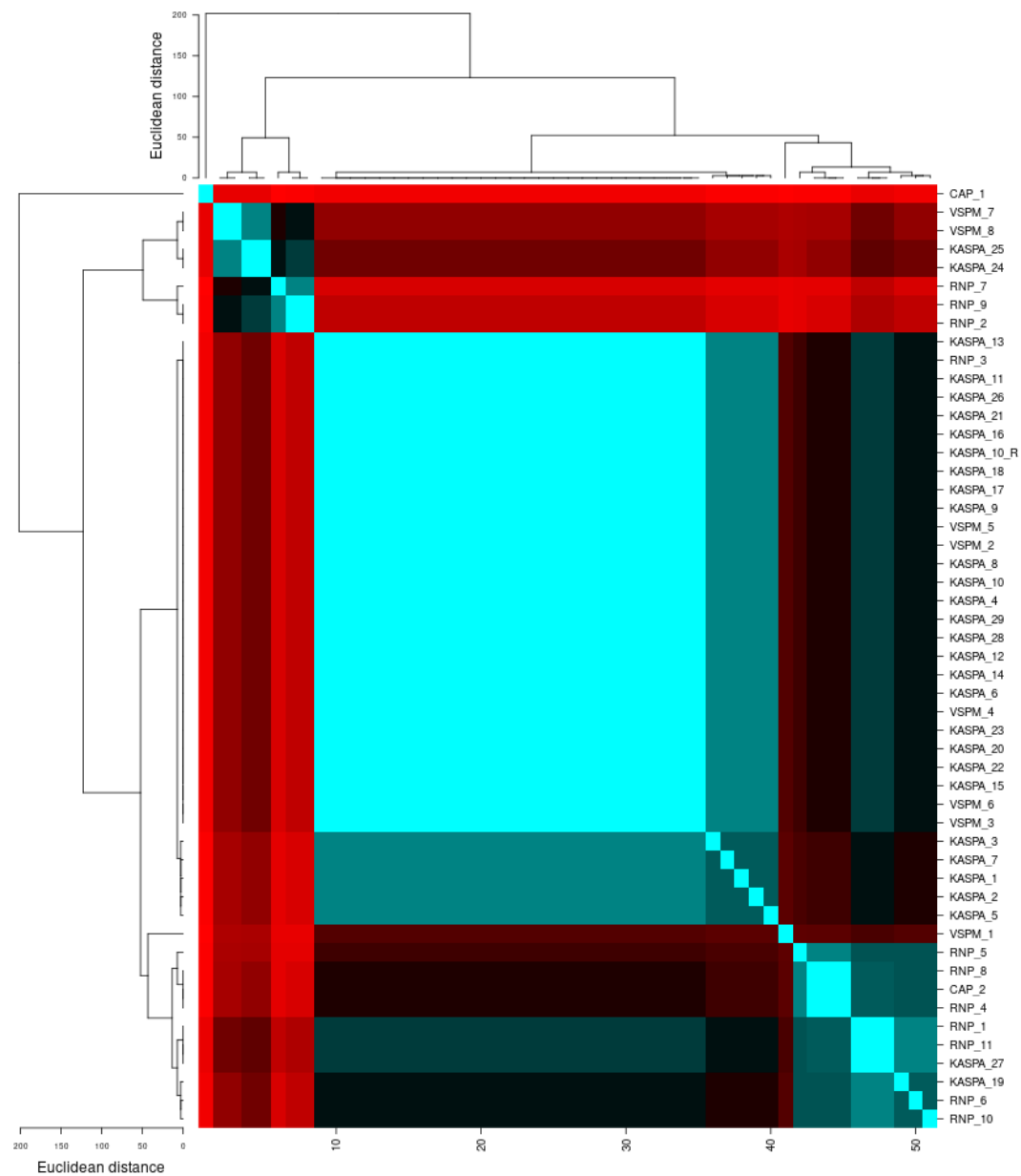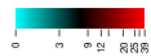

Supplement: Supplementary file 1 [file tpmd210593.SD1.pdf]
